# Effect of Partial H_2_O-D_2_O Replacement on the Anisotropy of Transverse Proton Spin Relaxation in Bovine Articular Cartilage

**DOI:** 10.1371/journal.pone.0115288

**Published:** 2014-12-29

**Authors:** Sirisha Tadimalla, Konstantin I. Momot

**Affiliations:** 1 School of Chemistry, Physics and Mechanical Engineering, Queensland University of Technology, Brisbane, Queensland, Australia; 2 Institute of Health and Biomedical Innovation, Kelvin Grove, Queensland, Australia; Dundee University, United Kingdom

## Abstract

Anisotropy of transverse proton spin relaxation in collagen-rich tissues like cartilage and tendon is a well-known phenomenon that manifests itself as the “magic-angle” effect in magnetic resonance images of these tissues. It is usually attributed to the non-zero averaging of intra-molecular dipolar interactions in water molecules bound to oriented collagen fibers. One way to manipulate the contributions of these interactions to spin relaxation is by partially replacing the water in the cartilage sample with deuterium oxide. It is known that dipolar interactions in deuterated solutions are weaker, resulting in a decrease in proton relaxation rates. In this work, we investigate the effects of deuteration on the longitudinal and the isotropic and anisotropic contributions to transverse relaxation of water protons in bovine articular cartilage. We demonstrate that the anisotropy of transverse proton spin relaxation in articular cartilage is independent of the degree of deuteration, bringing into question some of the assumptions currently held over the origins of relaxation anisotropy in oriented tissues.

## Introduction

Spin relaxation of water protons is a useful marker of the microstructure and composition of biological tissues [1], [2], including articular cartilage [3]–[5]. Proton spin relaxation is determined by intra- and intermolecular interactions of spins with the magnetic moments of neighbouring nuclei. These interactions are mediated by the dynamics of the water molecules, which in turn depend on the biopolymeric composition and cellular organisation of the tissue. Furthermore, in partially aligned tissues such as articular cartilage, relaxation rates can depend on the orientation of the tissue relative to the applied magnetic field (**B**_0_). This dependence (the so-called relaxation anisotropy) can be used as a probe of the three-dimensional architecture of the tissue at the microscopic level and can inform the interpretation of Magnetic Resonance Imaging (MRI) studies of cartilage biomechanics [6]–[8], complementing other MRI [9]–[11] and non-MRI [12] techniques.

Spin relaxation refers to the return of an excited spin magnetisation to its equilibrium state. The relaxation of water protons can be described by two simultaneously occurring processes – longitudinal relaxation (characteristic time T_1_) and transverse relaxation (characteristic time T_2_), which represent the return of the longitudinal and the transverse component of the magnetisation vector, respectively. It is convenient to characterise spin relaxation using spin relaxation rates, R_1_ = 1/T_1_ and R_2_ = 1/T_2_, rather than the relaxation times T_1_ and T_2_. Both relaxation processes are caused by interactions of the magnetic dipole of the nuclear spin with those of the neighbouring nuclei. This interaction is inherently stochastic, as it is modulated by molecular tumbling and translation and is dependent upon intermolecular collisions, van der Waals binding, and proton exchange with other molecules. Longitudinal relaxation is affected only by the fast motions at the frequency components of the order of ω_0_ and 2ω_0_, where ω_0_ is the resonant (Larmor) frequency [13]. Transverse relaxation is additionally sensitive to slow motions at the near-zero frequency components, which result in the rapid loss of phase coherence of the transverse magnetization without affecting the relative populations of the spin states [14].

Articular cartilage is a biological tissue mainly composed of Type II collagen (about 10% to 20% of the wet weight), proteoglycans (10% to 15% of the wet weight) and water (65% to 80% of the wet weight) [15]–[17]. Collagen forms an organized network of cross-linked fibres, which confines the hydrophilic proteoglycan aggregates covalently linked to it. The network can be schematically divided into three different zones based on differences between collagen fibre orientations across the depth of the cartilage tissue. The superficial zone is the thinnest zone and lies at the articular surface and contains collagen fibres oriented parallel to the surface. Next is the transitional zone, where collagen fibres are oriented randomly and lack predominant alignment. The radial zone is closest to the bone and contains fibres that are aligned almost perpendicular to the articular surface [16]. In addition to fibre orientation, the concentrations of macromolecules vary significantly with depth, with the highest amount of collagen and proteoglycans present in the radial zone and the lowest in the superficial zone [15].

Typically, water relaxation behaviour in articular cartilage and other biological tissues can be understood in terms of rapid chemical exchange between different pools of water – a slowly relaxing ‘free’ pool where water molecules are mobile, and a fast relaxing ‘bound’ pool where water molecules are hydrogen bonded to relatively immobile macromolecules. The exchange rate is fast on the MRI time-scale, so the apparent relaxation rate observed in spin-echo MRI is the weighted average of the relaxation rates in these two pools [7], [18], [19]:
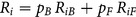
(1)where *i* = 1 or 2 for longitudinal and transverse spin relaxation, respectively, and B and F refer to the “bound” and “free” pools, respectively. Estimates of relaxation rates in different pools of water have been obtained using either multi-component data analysis or magnetisation transfer techniques [20], [21].

In articular cartilage, motion of water molecules ‘bound’ to collagen fibres is restricted by the alignment of the fibres and the orientation of these fibres with respect to the static field, **B_0_** significantly influences proton spin relaxation. It is well established that while longitudinal relaxation (T_1_) is orientation independent, transverse relaxation (T_2_) is strongly orientation dependent, or anisotropic [17]. This orientation dependence is clearly seen in T_2_-weighted images of articular cartilage oriented at different angles with respect to **B_0_** [22]. The depth profiles of transverse relaxation rates reveal a consistent pattern of low R_2_ values in the transitional zone and high R_2_ values in the radial zone. This depth-dependence shows up as bright and dark bands in T_2_-weighted images, demonstrating the influence of collagen fibre orientation on T_2_ relaxation [23], [24].

T_2_ anisotropy in collagen-rich tissues such as cartilage and tendon is usually attributed to the non-zero averaging of intra-molecular dipolar interactions due to the preferential alignment of water molecules bound to oriented collagen fibres [25], [26], resulting in residual dipolar couplings. The dipole-dipole interaction Hamiltonian, 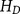, involving multiple interacting spins can be represented as a sum of all the pairwise interactions [27]:
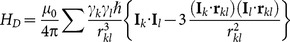
(2)where 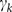 and 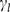 are the magnetogyric ratios of the coupled pair of nuclei, 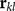 is the distance between spins *k* and *l*, and 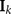 and 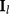 are the corresponding spin operators. The pairwise dipolar coupling constant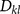, which shows the strength of the interaction, is then defined as
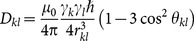
(3)where 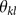 is the angle between the interproton vector and the main magnetic field **B_0_**. At an angle 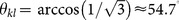, also called the magic angle, the strength of the dipolar interaction becomes zero.

Xia et al examined the R_2_ relaxation rates at several different values of 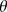, where 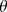 was the orientation of the normal to the articular surface with respect to **B_0_** [22]. They found that the R_2_ values *at all depths* from the articular surface qualitatively followed the 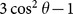 curve, attaining their minimum value at the magic angle orientation. Therefore, even with no knowledge of the distribution of collagen fibre orientations within the sample, the transverse relaxation rate, R_2_, can be empirically expressed as a sum of the isotropic and anisotropic contributions:
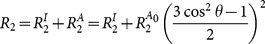
(4)where 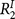 and 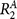 are the isotropic and anisotropic relaxation rates, respectively [26]. When 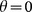, the anisotropic contribution attains its maximum amplitude, 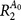. At the magic angle, 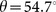, the anisotropic contribution is reduced to zero, the transverse relaxation rate attains its minimum value, is purely isotropic and equal to 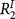. Equation (4) is valid at all depths from the articular surface and can, therefore, be used to estimate the isotropic and anisotropic contributions to transverse relaxation rates in cartilage with no prior knowledge of the collagen fibre orientation distribution.

One way to manipulate the contributions of dipolar interactions to proton spin relaxation in a sample is to replace some of the water with deuterium oxide, thereby creating an equilibrium mixture of H_2_O, D_2_O and HDO. As the magnetogyric ratio of deuterium 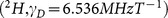 is approximately 7 times weaker than that of proton nucleus 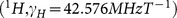, dipolar interactions are weaker and their contribution to relaxation can be expected to be reduced. Indeed, in HDO solutions of increasing concentrations of deuterium, both R_1_ and R_2_ relaxation rates exhibited a negative linear relationship with D_2_O concentration [28], [29].

The effect of deuteration on relaxation rates in cartilage is expected to be similar. Both the isotropic and anisotropic contributions to transverse relaxation are expected to be affected, although, the behaviour, in particular, of the anisotropic contribution to T_2_ relaxation is of foremost interest to us. In this paper, we present the results from our study of proton spin relaxation behaviour in bovine articular cartilage in the presence of deuterium oxide. We examined the longitudinal relaxation rates and the isotropic and anisotropic contributions to transverse relaxation rates, and their response to increasing levels of deuteration in cartilage. We anticipated significant decrease in the relaxation rates with increasing deuterium concentrations. Our results revealed unexpected and surprising behaviour of the anisotropic contribution to transverse relaxation, contradictory to the current understanding of the origins of relaxation anisotropy in ordered tissues.

## Methods

Bovine knee joints were obtained from a local abattoir (Teys Australia, Beenleigh, QLD 4207, Australia) and frozen 2 hours after slaughter. The use of bovine tissue was approved by the University Animal Ethics Committee, Queensland University of Technology (Approval no. 1200000211). The study was exempted from further ethics review as the tissue was obtained as a by-product of a commercial enterprise.

Normal patellae were later identified by visual examination. Four cylindrical plugs, of diameter 1 cm, were excised from different patellae with an intact layer of subchondral bone. The thickness of the cartilage layer was typically around 1.5 mm. The samples were placed in Phosphate buffered saline (PBS) prepared from PBS concentrate sachets (pH 7.4, NaCl 0.138M, KCl 0.0027M; Sigma-Aldrich, Australia). Protease inhibitors (Sigma-Aldrich, Australia) and 0.5 mg/mL sodium azide, NaN_3_ (Sigma-Aldrich, Australia) were added to the PBS in order to inhibit metalloproteinase activity and bacterial growth, respectively. A scalpel nick was made on the cartilage surface in order to consistently identify the same absolute location of the imaging slice during each imaging experiment.

Three D_2_O-containing PBS solutions were prepared by mixing 99.9% D_2_O (Sigma-Aldrich, Australia) with the PBS solution (prepared as discussed earlier). 0.371 g, 1.112 g, and 3.336 g of 99.9% D_2_O were added per 1 mL of the pure H_2_O-based PBS solution, achieving D_2_O molar ratios of 24.94%, 49.94%, and 74.92%, respectively. At D_2_O molar fractions significantly greater than 75%, the signal-to-noise ratio of ^1^H MR images was insufficient for reliable measurement of the ^1^H relaxation rates at the spatial resolution used. The three D_2_O-containing solutions will be hereafter referred to as 25%D_2_O, 50%D_2_O and 75%D_2_O solutions, respectively, and collectively as the D_2_O-PBS solutions. The PBS solution without any added D_2_O will be referred to as the H_2_O-PBS solution.

All MR measurements were performed at room temperature on a Bruker Avance nuclear magnetic resonance (NMR) spectrometer with a 7.0T vertical bore superconducting magnet equipped with a Micro2.5 micro-imaging probe and a 1.1 T/m triple-axis gradient set. A 15 mm birdcage RF coil was used.

### NMR Spectroscopy

As a gold standard of relaxation measurements, spectroscopic relaxation rate measurements were carried out on the H_2_O-PBS and D_2_O-PBS solutions without any cartilage sample present. An inversion recovery experiment (64 repetition times, TI: 10 to 100000 ms; approximately exponentially spaced) was used for T_1_-weighted measurements. A Hahn echo experiment and a Carr-Purcell-Meiboom-Gill (CPMG) experiment (64 echo times, TE: 2 to 20000 ms; spaced exponentially till 3200 ms and linearly spaced thereon) were performed to obtain two sets of T_2_-weighted measurements. The longitudinal relaxation rate, R_1_ = 1/T_1_, was determined by fitting an inversion recovery curve to the T_1_ data:
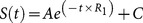
(5)where *t* is the inversion time, TI, *C* is the amplitude of the fully recovered signal, including noise, and *A+C* is the signal immediately after the inversion pulse. The three-parameter fit used in Equation (5) accounts for any errors due to imperfect RF pulses.

The transverse relaxation rate, R_2_ = 1/T_2_, was determined by fitting an exponential decay curve to the T_2_ data at each echo time TE:
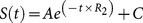
(6)where *t* = TE is the echo time, *A* is the maximum amplitude of the signal and *C* is noise.

### T1- and T2-weighted MRI

For the imaging experiments, the cartilage samples were placed in a 15 mm NMR tube (Wilmad, USA). Custom-made Teflon plugs placed at the bottom of the tube were used to orient the sample at the required angle. A correspondingly shaped Teflon plug placed on top of the sample prevented its movement during imaging. For each sample studied, imaging was first carried out in H_2_O-PBS. Images were obtained at two sample orientations θ_AS_ = 0° and θ_AS_ = 55°, where θ_AS_ was defined as the angle between the static magnetic field **B**_0_ and the normal to the articular surface of the sample. The sample was then equilibrated in the 25%D_2_O solution for at least 12 hours before imaging. Previous studies have shown that when equilibrated with 100% D_2_O, a>90% H_2_O/D_2_O replacement in all regions of the cartilage sample was achieved in 2 hours [30], [31]. Therefore, the equilibration time of 12 hours was deemed sufficient for a near-complete isotopic equilibration between the cartilage sample and the surrounding solution. The equilibrating solution was also used as the imaging medium to maintain hydration of the sample. Imaging was carried out at the two sample orientations and this process was repeated with the remaining two D_2_O solutions, resulting in 8 sets of images for each sample (4 D_2_O concentrations × 2 orientation angles). Imaging parameters were as follows: 256×256 matrix size, 30 mm×30 mm field of view (FOV), in-plane resolution 117.2×117.2 µm and 0.5 mm slice thickness. Image reconstruction was performed in Paravision software using magnitude Fourier transformation of the MR data.

The T_1_ series of images were acquired using Bruker multi-slice multi-echo (MSME) spin-echo based imaging sequence with a series of user-defined repetition times (TR). Up to 36 repetition times ranging from 200 to 9000 ms (approximately linearly spaced) and two echo times (TE), 7 and 14 ms, were used. Relaxation rate maps were constructed by fitting the variation of the signal amplitude in the respective series of images for the individual voxels in the image. R_1_ values were determined by fitting a saturation recovery curve to the T_1_ data. The mathematical equation describing the curve was identical to Equation(5), but the meaning of the parameters was different:*t* = TR was the repetition time, *C* was the amplitude of the fully recovered signal, including noise, and *A+C* was the amplitude of the fully saturated signal. Only the T_1_ images obtained with TE = 7 ms were used, as they showed the least amount of ghosting artifacts.

The T_2_ series of cartilage images were acquired using a Bruker multi-slice multi-echo spin-echo (MSME) based sequence with a single repetition time (TR) of 4000 ms and 60 equidistantly spaced echo times (TE) ranging from 8 ms to 480 ms. R_2_ relaxation rate maps were obtained by fitting Equation(6) to the MSME relaxation curve. All fits were performed using a Levenberg-Marquardt based non-linear least squares algorithm. In some regions of the cartilage, the R_2_ relaxation rates were very high, resulting in the decay of the MR signal on a time scale faster than the TE spacing (8 ms). In such cases, the decay curve did not support a reliable relaxation fit, and the relevant voxel was excluded from the analysis. Acceptance criteria for estimated relaxation rates were therefore defined as: R_1_>0.0001 ms^−1^, R_1_<0.002 ms^−1^, R_2_>0.002 ms^−1^, and R_2_<0.125 ms^−1^ to ensure that only physically meaningful relaxation rates were included in further analysis.

### Data analysis

For each of the four samples imaged, 16 imaging datasets were obtained (eight T_1_-weighted and eight T_2_-weighted datasets, corresponding to four D_2_O concentrations and two θ_AS_ values). Correspondingly, eight R_1_ relaxation rate maps and eight R_2_ maps were obtained. Imaging experiments were also performed on the D_2_O-PBS solutions in the absence of cartilage using the same parameters as described earlier, with TR = 40 to 90000 ms for T_1_-weighted imaging and TR = 14000 ms for T_2_-weighted imaging respectively being the only changes. R_1_ and R_2_ relaxation rate maps were calculated using equations (5) and (6) respectively.

**Figure 1:**
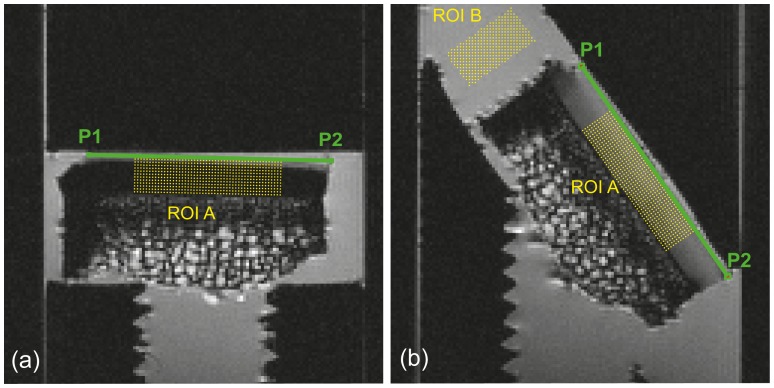
Regions of interest ROI A and ROI B are shown on a R_1_ relaxation map of a sample. P1 and P2 are the manually selected endpoints of the articular surface represented by the green line at the two sample orientations (a) θ_AS_ = 0° and (b) θ_AS_ = 55°. The rectangular voxel array within ROI A was created using this line as reference, while ROI B was manually selected.

A region of interest, labelled ROI A, was selected within the cartilage. A T_2_-weighted image with the greatest contrast between cartilage and solution was chosen at each orientation and at each D_2_O concentration as a reference data set. Two points were manually selected at the ends of the cartilage surface in the image. A line drawn between these points was identified as the surface, the depth of the cartilage was estimated, and equidistant voxels on either side of the midpoint of the line segment were obtained to form a rectangular region of interest as shown in Fig. 1. This process was repeated for images at all the D_2_O concentrations at both the sample orientations. The voxel arrays constituting this region were then comparable across all images and relaxation maps of the sample data set. Another region of interest, labelled ROI B, was manually selected in the imaging solution near the cartilage sample in R_1_ and R_2_ relaxation maps at θ_AS_ = 55°, as shown in Fig. 1. Regions subject to magnetic susceptibility artifacts were avoided.

Based on Equation(4), the isotropic contribution was defined as the R_2_ value at the magic angle. The anisotropic contribution was then calculated as the difference between the R_2_ relaxation rates at θ_AS_ = 0° and θ_AS_ = 55°. In voxels with an isotropic distribution of collagen fibres, it is expected that the R_2_ values measured at θ_AS_ = 0° would be equal to those measured at θ_AS_ = 55°. However, the finite noise level resulted in some voxels exhibiting R_2_ values that were slightly smaller at θ_AS_ = 0° than at θ_AS_ = 55°, resulting in an anisotropic contribution that was apparently negative but not statistically different from zero. Additionally, at θ_AS_ = 0°, the MR signal in some voxels, especially in the radial zone, was too low to produce a reliable fit. These voxels were assigned an R_2_ value of zero, so the calculated anisotropic contribution appeared to be negative. As negative anisotropic contributions have no physical meaning, all voxels which exhibited a negative difference (up to 30% of the total cartilage voxels) were removed from further analysis. Means and standard deviations of the two contributions were obtained at each depth from the articular surface from a concatenated array of measurements from ROI A in each sample. The procedure was repeated for the corresponding ROIs in relaxation rate maps obtained at the remaining three D_2_O concentrations. Means and standard deviations were also obtained in manually selected regions of interest in relaxation rate maps of the D_2_O-PBS solutions obtained in the absence of cartilage samples. All image post-processing and data analysis methods were implemented in Matlab (R2012b, The Mathworks, Natick, MA, USA) using custom-written code.

## Results

### Longitudinal and transverse relaxation rates in solution

#### Pure solution

**Figure 2:**
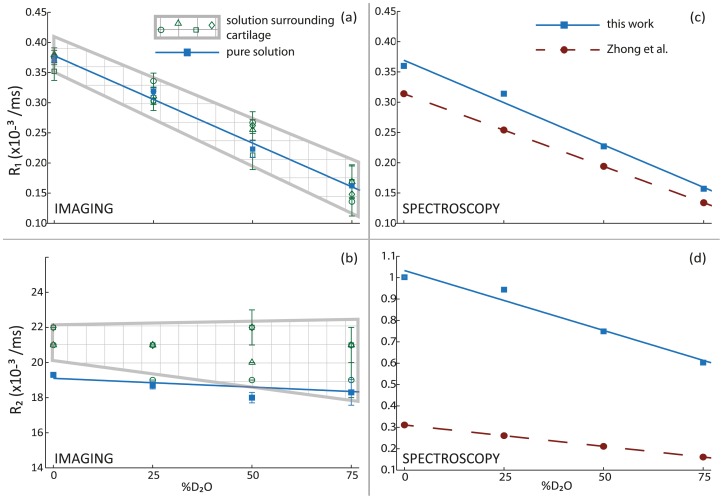
Effect of deuteration on proton relaxation rates in solution. Apparent (a) R_1_ rates and (b) R_2_ rates in pure solution (blue squares; solid blue line) and in solution surrounding cartilage samples (multiple green symbols; checkered region) obtained using imaging measurements. Spectroscopic measurements of (c) R_1_ rates and (d) R_2_ rates in pure solution obtained using inversion-recovery and CPMG experiments respectively in the present study (blue squares; solid blue line) and the corresponding values obtained by Zhong et al. (red circles; broken red line).

Longitudinal and transverse relaxation rates were measured in the D_2_O-PBS and H_2_O-PBS solutions using both spectroscopic and imaging experiments. The longitudinal (R_1_) relaxation rates exhibited a negative linear relationship with deuteration levels. This relationship can be seen in Fig. 2a and 2c, which depict the apparent R_1_ values observed in imaging and spectroscopic measurements, respectively. The parameters of the respective linear least-squares fits are shown in Table 1. Row 2 in Table 1 shows the fit parameters for the apparent R_1_ values from imaging measurements (solid blue line in Fig. 2a); row 1i in Table 1 shows the fit parameters for the inversion-recovery spectroscopic R_1_ values (solid blue line in Fig. 2c). Also shown in Fig. 2c is the R_1_ data obtained by Zhong et al (red broken line) using spectroscopic inversion-recovery measurements [28]; row 1iv in Table 1 shows the fit parameters for this set of R_1_ data. The magnitudes of the spectroscopic R_1_ values obtained in this work and by Zhong et al are within the range of apparent R_1_ values obtained using imaging measurements.

**Table 1:** Proton R_1_ and R_2_ as a function of %molar concentration of D_2_O in the D_2_O-PBS and H_2_O-PBS solutions fitted using least-squares algorithm.

Experiment		R_1_ (×10^−3^ ms^−1^)	R_2_ (×10^−3^ ms^−1^)
**Spectroscopy – pure solution**	i. Inversion recovery experiment(Fig. 2c)	−0.28d+0.3693	
	ii. CPMG experiment (Fig. 2d)		−0.56d+1.0333
	iii. Hahn spin echo experiment		−1.38d+3.8687
	iv. Zhong et al results (Fig. 2c and 2d)	*Inversion recovery*-0.24d+0.3139	*CPMG*-0.20d+0.3110
**Imaging – pure solution**		−0.29d+0.3779	–
**Imaging – in the presence of cartilage samples**	i. Sample #1	−0.29d+0.3817	–
	ii. Sample #2	−0.26d+0.3551	–
	iii. Sample #3	−0.28d+0.3823	–
	iv. Sample #4	−0.31d+0.3963	–

The % molar concentration of D_2_O is denoted by d and is expressed as a fraction. For example, at 25% molar concentration of D_2_O, d = 0.25.

A similar negative linear relationship was observed for the R_2_ relaxation rates measured spectroscopically, as shown in Fig. 2d. R_2_ relaxation rates obtained spectroscopically using CPMG experiments, in this work (blue squares; solid blue line) and by Zhong et al. (red circles; broken red line) [29]), are shown. In the spectroscopic R_2_ dataset, the apparent R_2_ values obtained using the Hahn spin echo experiments were several times greater than the R_2_ values obtained using the CPMG experiments. Due to the large disparity between the two R_2_ values, the R_2_ values measured using the Hahn spin echo sequence were not included in Fig. 2d. The parameters of the linear least-squares fits for the Hahn spin echo R_2_ dataset and the CPMG R_2_ dataset obtained in this work, and the CPMG R_2_ dataset obtained by Zhong et al. are shown in rows 1iii, 1ii and 1iv, respectively.

The mean apparent R_2_ values obtained from the imaging measurements are shown in Fig. 2b (blue squares and solid blue line). These values were approximately two orders of magnitude greater than the R_2_ values obtained spectroscopically. The mean apparent R_2_ values obtained from the imaging measurements also did not exhibit any significant dependence on deuteration. Linear least-squares fits of the apparent imaging R_2_ values, therefore, were trivial and are not presented here.

#### Solution surrounding an articular cartilage sample

Imaging experiments were carried out to measure longitudinal and transverse relaxation rates in the same D_2_O-PBS and H_2_O-PBS solutions, with each of the four articular cartilage samples immersed in the solutions. As described in the Methods section, a region of interest (exemplified by ROI B in Fig. 1) was defined in the solution part of each image. The means and the standard deviations of the apparent R_1_ and R_2_ values were obtained in this region.

The apparent R_1_ values obtained in the solution in the presence of each of the cartilage samples are shown in Fig. 2a (open green symbols and checkered region). The parameters of the corresponding linear least-squares fits are shown in rows 3i-iv in Table 1. It is clear that the negative linear relationship between the apparent R_1_ values and deuteration levels remained unaffected by the presence of cartilage samples.

The apparent R_2_ values obtained in the solutions in the presence of each of the cartilage samples are shown in Fig. 2b (open green symbols and checkered region). These R_2_ values did not exhibit any significant change with deuteration, and therefore, the corresponding linear least-squares fits were trivial and are not shown here (rows 3i-iv in Table 1). The magnitudes of these R_2_ values were comparable to the apparent R_2_ values measured in pure solutions using imaging measurements (blue squares and solid blue line in Fig. 2b).

### Proton spin relaxation in articular cartilage

For each cartilage sample imaged, a region of interest (exemplified by ROI A in Fig. 1) was defined to encompass cartilage voxels, as described in the Methods section. The means and the standard deviations of the apparent longitudinal relaxation rates and the isotropic and anisotropic contributions to the apparent transverse relaxation rates in this region were obtained as described earlier.

#### Longitudinal relaxation

**Figure 3:**
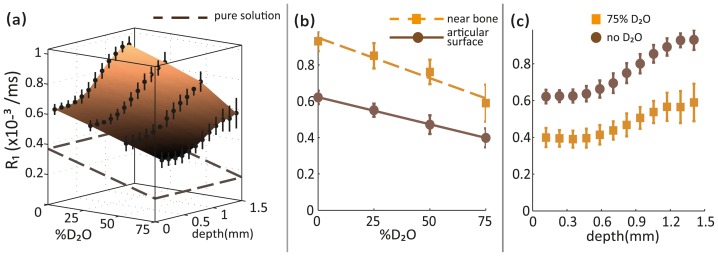
Effect of deuteration on longitudinal relaxation rates in articular cartilage. (a) A surface plot showing the variation of apparent R_1_ values in articular cartilage with deuteration and depth from articular surface. The plane formed by the broken lines represents the apparent mean R_1_ values measured (using imaging) in the H_2_O-PBS and D_2_O-PBS solutions surrounding all the cartilage samples. (b) Negative linear dependence of R_1_ values on deuteration at the articular surface (brown circles; solid brown line) and near the bone (orange squares; broken orange line) and (c) Depth-dependence of R_1_ values before deuteration (brown circles) and at 75% deuteration (orange squares) is shown.

The apparent R_1_ values in cartilage varied significantly with both deuteration and depth from articular surface. As shown in the three-dimensional plot in Fig. 3a, the R_1_ values demonstrated a negative linear relationship with deuteration. This relationship was similar to the negative linear relationship observed between the apparent R_1_ values and the deuteration level in the H_2_O-PBS and D_2_O-PBS solutions surrounding the cartilage samples, obtained using imaging. The plane formed by the dashed brown lines in Fig. 3a represents the mean R_1_ values measured (using imaging) in the H_2_O-PBS and D_2_O-PBS solutions surrounding all the cartilage samples. The variation of the apparent R_1_ values with deuteration in articular cartilage (measured using imaging) was uniform across the depth of the cartilage. In Fig. 3b, the apparent R_1_ relaxation rates in cartilage at the articular surface and near the bone are depicted as the solid brown line fitted to the solid brown circles and the dashed orange line fitted to the solid orange squares, respectively. It was clear that the apparent R_1_ values in cartilage near the bone were significantly larger compared to those at the articular surface. In the surface plot in Fig. 3a, it was also evident that there was a clear pattern in the depth-dependence of R_1_ values, and the degree of variation of the R_1_ rates across the depth of cartilage was comparable at different levels of deuteration. Fig. 3c shows the depth profiles of R_1_ values in cartilage before deuteration (solid brown circles) and at 75% deuteration (solid orange squares). While the increase in R_1_ values with deuteration was obvious, the shapes of the depth profiles at the two deuteration levels were almost identical.

#### Isotropic contribution to transverse relaxation

**Figure 4:**
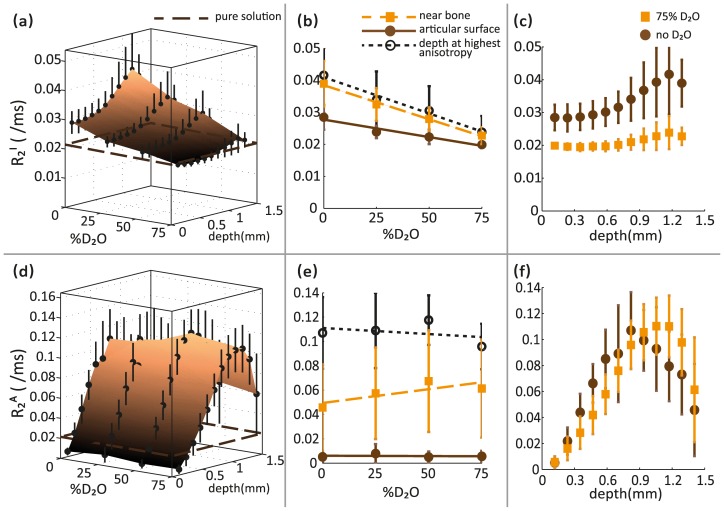
Effect of deuteration on the isotropic and anisotropic contributions to transverse relaxation rates in articular cartilage. Surface plots showing variation of the (a) isotropic (R_2_^I^) and (d) anisotropic (R_2_^A^) contributions to transverse relaxation in articular cartilage with deuteration and depth from articular surface. Apparent mean R_2_ values measured (using imaging) in the H_2_O-PBS and D_2_O-PBS solutions surrounding all the cartilage samples are represented by the plane formed by the broken brown lines. Separate 2D plots show the effects of deuteration on (b) R_2_^I^ and (e) R_2_^A^ values at the articular surface (solid brown circles; solid brown line), near the bone (orange squares; broken orange line), and at the depth of highest calculated anisotropy (open black circles; dotted black line). Depth-dependence of (c) R_2_^I^ and (f) R_2_^A^ values before deuteration (brown circles) and at 75% deuteration (orange squares) is also shown.

As shown in Fig. 4a, the isotropic contribution to the apparent transverse relaxation rate, denoted by R_2_^I^, varied significantly with both deuteration and depth from articular surface. R_2_^I^ values demonstrated an almost negative linear relationship with deuteration level, unlike the apparent imaging R_2_ values measured in the H_2_O-PBS and D_2_O-PBS solutions surrounding the cartilage samples. The plane formed by the dashed brown lines in Fig. 4a represents the mean apparent R_2_ values measured (using imaging) in the H_2_O-PBS and D_2_O-PBS solutions surrounding all the cartilage samples.

A substantial decrease in R_2_^I^ values with increasing deuteration was observed at all depths from the articular surface as shown in Fig. 4b. In this figure, the R_2_^I^ values at the articular surface, near the bone and at the depth of the highest calculated anisotropy in the cartilage are represented by the solid brown line fitted to the solid brown circles, the broken orange line fitted to the solid orange squares, and the dotted black line fitted to the open black circles, respectively. It is evident from this figure that R_2_^I^ values nearer to the bone were greater than those measured at the articular surface. The depth-dependence of the R_2_^I^ values at different deuteration levels is depicted in Fig. 4c. A clear pattern of R_2_^I^ values across the depth of the cartilage was observed before deuteration (solid brown circles) and at 75% deuteration (solid orange squares).

#### Anisotropic contribution to transverse relaxation

As shown in Fig. 4d, the anisotropic contribution to transverse relaxation rate, denoted by R_2_^A^, varied significantly with depth from articular surface as expected, but was almost unaffected by deuteration. This independence of R_2_^A^ values of deuteration is very similar to that exhibited by the apparent R_2_ values in the H_2_O-PBS and D_2_O-PBS solutions surrounding the cartilage samples measured using imaging. It should be noted, however, that the absolute R_2_^A^ values were several times greater than the apparent R_2_ values measured in the H_2_O-PBS and D_2_O-PBS solutions. The plane formed by the broken brown lines in Fig. 4d represents the mean apparent R_2_ values measured in the H_2_O-PBS and D_2_O-PBS solutions surrounding all the cartilage samples.

Fig. 4e shows the R_2_^A^ values at different deuteration levels at three different depths from the articular surface. The R_2_^A^ values at the articular surface, near the bone and at the depth of highest calculated anisotropy in cartilage are represented by the solid brown line fitted to solid brown circles, the broken orange line fitted to the solid orange squares, and the dotted black line fitted to the open black circles, respectively. The R_2_^A^ values were independent of the deuteration level at all depths from the articular surface of the cartilage. Similarly, as shown in Fig. 4f, the depth-dependence of R_2_^A^ values is also unaffected by deuteration. The depth profiles of the R_2_^A^ values before deuteration (solid brown circles) and at 75% deuteration (solid orange squares) do not exhibit any significant differences.

## Discussion

### Effects of deuteration on spin relaxation rates

Deuteration has been effectively used to reduce longitudinal and transverse relaxation rates of protons in solutions [13], [28], [29], [32]–[35]. In solutions, we, and others, observed a negative linear relationship between R_1_ rates and %D_2_O. This relationship is independent of the measurement method as well as of the possible presence of leached proteoglycans from the cartilage sample. In articular cartilage, the negative linear relationship between R_1_ rates and %D_2_O remained the same, but the absolute values of the relaxation rates were significantly greater than in solutions. This is expected because in biological tissues, water molecules spend a significant fraction of time hydrogen-bonded to the large, immobile macromolecules, resulting in reduced molecular mobility and more efficient relaxation of water protons. R_1_ rates also increased significantly with depth from articular surface at all deuteration levels, and the effect of deuteration was not the same at all depths from the articular surface. This is likely due to the increasing concentrations of collagen and proteoglycan macromolecules towards the deeper zones of the cartilage. As demonstrated by Zhong et al, the cross-relaxation effects that influence R_1_ rates are independent of deuteration, but are significantly affected by the molecular weights of proteins in solution. In both the solutions (Fig. 2) and in cartilage (Fig. 3), we found that the relationship between R_1_ rates and deuteration is very similar to that observed by Zhong et al., suggesting that proton-proton dipolar interactions within and between water molecules are the primary source of longitudinal relaxation.

Dipolar interactions are also a significant source of transverse relaxation, indicated by the negative linear relationship observed between spectroscopically obtained R_2_ rates and %D_2_O in solutions. Although this relationship was also reported by Zhong et al in pure H_2_O/D_2_O solutions, our absolute R_2_ values were significantly larger (Fig. 2d). A possible reason for this could be the presence of phosphate ions and dissolved oxygen in the H_2_O-PBS and D_2_O-PBS solutions that we studied. More interestingly, the R_2_ values measured using imaging experiments were even larger and did not exhibit any relationship with deuteration (Fig. 2b). This is a consequence of using a spin-echo imaging sequence, where diffusion induced signal loss can contribute heavily to calculated R_2_ rates. The signal loss arises due to spins moving via diffusion during the imaging gradient. Any effect of deuteration on the apparent transverse relaxation rates measured in solutions is therefore masked by the extremely large influence of diffusion. In articular cartilage, it is only to be expected that the transverse relaxation rates are larger compared to those in the H_2_O-PBS and D_2_O-PBS solutions. The reduced mobility of water molecules ‘bound’ to macromolecules, along with chemical exchange between ‘free’ and ‘bound’ water and other slow processes result in faster relaxation. Diffusion effects were, however, ignored because the intrinsic relaxation rates in articular cartilage are typically much greater than in solutions.

### Isotropic and anisotropic contributions to transverse relaxation in cartilage

Unlike the R_1_ values illustrated in Fig. 3, the dependence of R_2_ values in articular cartilage on the degree of deuteration varied significantly with depth from the articular surface. For example, in the radial zone (depth ∼1 mm) at the perpendicular orientation, the R_2_ value was found to be 0.042±0.063 ms^−1^ in H_2_O-PBS and 0.096±0.063 ms^−1^ in 75% D_2_O-PBS, indicating no significant difference, while in the transitional zone (depth ∼0.5 mm), the R_2_ value was found to be 0.096±0.014 ms^−1^ in H_2_O-PBS and 0.062±0.014 ms^−1^ in 75% D_2_O-PBS, resulting in a p-value <0.01 obtained using a Student t-test.

**Figure 5:**
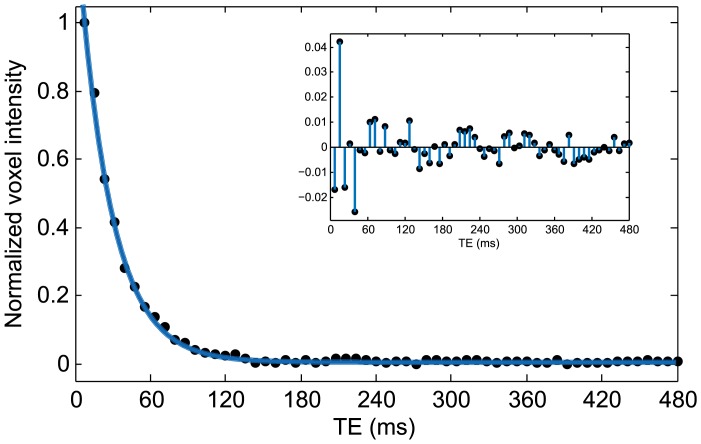
T_2_-weighted signal intensities in a representative voxel at multiple echo times. At the minimum echo time used in our study, the shorter T_2_ components would have already decayed. The data points are, therefore, fit to a mono-exponential decay curve as shown. Figure inset shows the residual deviations of the data points from the fit.

Therefore, further insight into the behaviour of the transverse relaxation rates can be obtained by considering their orientational dependence. Using the R_2_ maps obtained at the two orientations of each cartilage sample, we were able to separate the observed R_2_ value into an isotropic and an anisotropic contribution, as described earlier. It is important to note that the isotropic and anisotropic contributions to transverse relaxation defined in this study describe bulk orientation-independent and orientation-dependent R_2_ values, respectively. These terms have no direct relation to the multiple components of T_2_ relaxation in cartilage and tendon observed by several researchers [20], [36]–[40] that are generally associated with the different molecular environments surrounding the water molecules. In our study, we used long echo times (minimum TE = 8 ms), which do not allow us to reliably measure multiple T_2_ components. As shown in Fig. 5, the decay of the T_2_-weighted signal in cartilage (in a representative voxel) at these long echo times is clearly mono-exponential. This is not disadvantageous to our investigation, however, as we are primarily concerned about the relative change in T_2_ anisotropy with deuteration.

### Contributions of dipolar interactions to transverse relaxation anisotropy

It is widely accepted that residual dipolar couplings arising from intramolecular dipolar interactions in ‘bound’ water molecules are the dominant mechanism behind transverse relaxation anisotropy in oriented tissues such as cartilage and tendon [40]–[44]. In theory, dipolar couplings arise from either intramolecular (between protons within the same molecule) or intermolecular (between protons in different molecules) dipolar interactions. From Equation(3), we know that the strength of the dipolar interaction is related to the distance between protons as **r** ^−**3**^. If **r** is small and molecular diffusion is isotropic, the dipolar interactions usually average to zero due to diffusion. In the case of water molecules bound to oriented collagen fibres, the dipolar interactions don’t average out and residual dipolar couplings are formed. If **r** is large, they average to zero only if there is no spatial variation of the sample magnetization. Whenever a magnetic field gradient is applied, as in an imaging experiment, intermolecular dipolar interactions cannot be ignored [27]. In addition, long-range dipolar interactions can also contribute to relaxation. However, the timescale of intermolecular interactions in connective tissues like cartilage and tendon is significantly longer than typically observed relaxation times, and their contributions to transverse relaxation are generally neglected [45], [46].

Eliav et al obtained DQF NMR spectra of tendon at two different deuteration levels and demonstrated that the change in the intensity of the spectra was clearly proportional to the change in the amount of H_2_O present within the samples. They replicated these results using other spectroscopic pulse sequences, leading to the conclusion that the dominant interaction was intramolecular [42]. These findings are consistent with other research on ^1^H and ^2^H spectra in oriented tissues which showed that the observed residual dipolar couplings originate predominantly from intramolecular dipolar interactions [41], [47]. They, however, did not consider the direct contributions of intramolecular dipolar interactions to T_2_ relaxation anisotropy. We, on the other hand, found that while the isotropic R_2_ contribution clearly decreased with increasing concentrations of D_2_O, the anisotropic R_2_ contribution remained unaffected (Fig. 4). If the residual dipolar couplings arising from intramolecular dipolar interactions were indeed the dominant mechanism for T_2_ anisotropy, a significant decrease in the anisotropic R_2_ contribution should have been observed after proton-deuterium replacement in our cartilage samples. In the absence of such an observation, it is important to reconsider intermolecular dipolar interactions and the role played by non-exchanging protons on the collagen fibres.

### An alternative hypothesis

Another explanation of T_2_ anisotropy in ordered collagenous tissues considers that intramolecular dipolar couplings combined with slow diffusional walk of the water molecules around the collagen fibre, mediated by chemical exchange are the dominant relaxation mechanism in bound water molecules [26], [41], [48]. In light of the new evidence presented in this study, intermolecular dipolar couplings of water molecule protons with the non-exchanging protons on collagen fibres may be more significant than previously considered. As the non-exchanging collagen fibre protons cannot participate in the deuteration process, the anisotropic contribution to transverse relaxation is then expected to be independent of deuteration. We hypothesize that these intermolecular dipolar couplings between water and biopolymer protons, along with slow diffusion of the water molecules around the collagen fibre modulated by chemical exchange are responsible for T_2_ anisotropy. Detailed investigations on our hypothesized and other possible models of ‘bound’ water molecule dynamics are necessary and may further our understanding of the mechanisms behind proton spin transverse relaxation anisotropy in articular cartilage and other partially aligned tissues.
